# Association of glucose homeostasis measures with heart rate variability among Hispanic/Latino adults without diabetes: the Hispanic Community Health Study/Study of Latinos (HCHS/SOL)

**DOI:** 10.1186/s12933-016-0364-y

**Published:** 2016-03-16

**Authors:** Michelle L. Meyer, Nathan M. Gotman, Elsayed Z. Soliman, Eric A. Whitsel, Raanan Arens, Jianwen Cai, Martha L. Daviglus, Pablo Denes, Hector M. González, Juan Moreiras, Gregory A. Talavera, Gerardo Heiss

**Affiliations:** University of North Carolina at Chapel Hill, 137 E. Franklin St, Suite 306, Chapel Hill, NC 27514 USA; Wake Forest School of Medicine, Winston Salem, NC USA; Division of Respiratory and Sleep Medicine, The Children’s Hospital at Montefiore, Albert Einstein College of Medicine, Bronx, NY USA; University of Illinois at Chicago College of Medicine, Chicago, IL USA; Northwestern University, Chicago, IL USA; Michigan State University, East Lansing, MI USA; University of Miami, Coral Gables, FL USA; San Diego State University, San Diego, CA USA

**Keywords:** Autonomic function, Insulin resistance, Type 2 diabetes mellitus, Parasympathetic and sympathetic nervous system, Cohort study

## Abstract

**Background:**

Reduced heart rate variability (HRV), a measure of cardiac autonomic function, is associated with an increased risk of cardiovascular disease (CVD) and mortality. Glucose homeostasis measures are associated with reduced cardiac autonomic function among those with diabetes, but inconsistent associations have been reported among those without diabetes. This study aimed to examine the association of glucose homeostasis measures with cardiac autonomic function among diverse Hispanic/Latino adults without diabetes.

**Methods:**

The Hispanic community Health Study/Study of Latinos (HCHS/SOL; 2008–2011) used two-stage area probability sampling of households to enroll 16,415 self-identified Hispanics/Latinos aged 18–74 years from four USA communities. Resting, standard 12-lead electrocardiogram recordings were used to estimate the following ultrashort-term measures of HRV: RR interval (RR), standard deviation of all normal to normal RR (SDNN) and root mean square of successive differences in RR intervals (RMSSD). Multivariable regression analysis was used to estimate associations between glucose homeostasis measures with HRV using data from 11,994 adults without diabetes (mean age 39 years; 52 % women).

**Results:**

Higher fasting glucose was associated with lower RR, SDNN, and RMSSD. Fasting insulin and the homeostasis model assessment of insulin resistance was negatively associated with RR, SDNN, and RMSSD, and the association was stronger among men compared with women. RMSSD was, on average, 26 % lower in men with higher fasting insulin and 29 % lower in men with lower insulin resistance; for women, the corresponding estimates were smaller at 4 and 9 %, respectively. Higher glycated hemoglobin was associated with lower RR, SDNN, and RMSSD in those with abdominal adiposity, defined by sex-specific cut-points for waist circumference, after adjusting for demographics and medication use. There were no associations between glycated hemoglobin and HRV measures among those without abdominal adiposity.

**Conclusions:**

Impairment in glucose homeostasis was associated with lower HRV in Hispanic/Latino adults without diabetes, most prominently in men and individuals with abdominal adiposity. These results suggest that reduced cardiac autonomic function is associated with metabolic impairments before onset of overt diabetes in certain subgroups, offering clues for the pathophysiologic processes involved as well as opportunity for identification of those at high risk before autonomic control is manifestly impaired.

## Background

Metabolic dysregulation is associated with reduced cardiac autonomic function [1] and is implicated in the development of cardiac autonomic neuropathy among those with type 2 diabetes [2, 3]. Cardiac autonomic function can be non-invasively measured by heart rate variability (HRV). Reduced HRV has been associated with cardiometabolic risk factors [4] and the metabolic syndrome [5–7], suggesting that autonomic dysfunction may occur before overt diabetes. In a murine model, diabetic and infarcted groups showed decreased HRV compared to controls [8].

Impairment in glucose homeostasis is associated with reduced autonomic function among people with diabetes [4, 9–12]; however, the association has not been sufficiently examined among those without diabetes [9, 13]. Prior studies did not include a comprehensive set of glucose homeostasis measures and were primarily among Caucasian samples. While Hispanic/Latino adults in the United States have a high burden of cardio metabolic risk factors [14], the metabolic syndrome [15], and high risk for diabetes [16], the association of glucose homeostasis measures and HRV has not been examined among Hispanic/Latino adults without diabetes.

The aim of this study, therefore, was to examine the associations of fasting insulin, fasting glucose, glycated hemoglobin (HbA_1c_), and insulin resistance with cardiac autonomic function as estimated by ultra-short term measures of HRV among participants without diabetes in the Hispanic Community Health Study/Study of Latinos (HCHS/SOL). Understanding factors associated with HRV among this population with a high burden of cardiometabolic risk could provide insight into mechanisms of reduced cardiac autonomic function and the potential to identify individuals at risk for metabolic disease before autonomic control is manifestly impaired.

## Methods

### Study cohort

HCHS/SOL is a multicenter epidemiologic cohort study examining chronic disease, risk and prevalence in 16,415 Hispanics/Latinos aged 18–74 years of Mexican, Central American, Cuban, Dominican, Puerto Rican, South American and other heritage groups. Details concerning the sampling strategy have been published elsewhere [17, 18]. Briefly, the HCHS/SOL target population was defined as all non-institutionalized self-identified Hispanic/Latino adults aged 18–74 years residing in defined geographical areas (census block groups) at the following four field centers: Bronx, NY, Chicago, IL, Miami, FL, and San Diego, CA. Participants were selected from the target population using a two-stage area household probability sampling approach. Over-sampling at both stages of sample selection was used to increase the likelihood that a selected address yielded an eligible household and to increase the proportion of participants ages 45–74 years (n = 9714, 59.2 %). All participants provided written informed consent, and the study was approved by the Institutional Review Boards at all field centers, coordinating center, central labs, and reading centers.

Participants on whom an electrocardiogram (ECG) was not performed (n = 183), had diabetes (n = 2868), were using antiarrhythmic medication or self-reported using medication for abnormal heart rhythm (n = 265), were age >75 years (n = 7), or were missing information on key variables (n = 277) were excluded from the analysis. Key variables included Hispanic/Latino background, smoking status, waist circumference (WC), body mass index (BMI), fasting insulin, fasting glucose, and HbA_1c_. Additionally, guided by the current recommendations for measurement of HRV [19], we also excluded participants not in sinus rhythm or with rhythm disorders (n = 693), and low quality ECGs to ensure the accuracy and quality of the HRV measures (n = 128). Thus, these analyses are based on data from 11,994 participants (52 % women).

### Data collection

Study examinations were conducted at field centers by certified study personnel following a standardized protocol. The details of the examination have been previously described [18]. In brief, participants were asked to fast, not smoke 12 h before the examination, refrain from vigorous physical activity the morning of the examination, and bring all prescription and nonprescription medication taken by them in the past 4 weeks. The medications were inventoried and therapeutically classified using a Master Drug Data Base (Medispan MDDB©) supplemented with Spanish-language brand and generic name equivalents from Lexi-Comp Online™ and OVID© Martindale. Study personnel administered questionnaires to collect medical history, demographic factors, education, income, country of origin, length of residence in the United States, language preference, and lifestyle information. Anthropometric measurements included body weight (kg), height (cm), WC and calculated BMI. Three sitting blood pressure measurements were taken with an automatic sphygmomanometer (OMRON HEM-907 XL, Omron Healthcare Co. Ltd., Kyoto, Japan) after a 5 min rest and the last two measurements were averaged. Blood and urine specimens were collected and shipped to the HCHS/SOL Central Laboratory to be assayed, i.e. for total cholesterol, high-density lipoprotein, low-density lipoprotein, triglycerides, and fasting glucose and insulin. Hypertension was defined as systolic blood pressure ≥140 mm/Hg, diastolic blood pressure ≥90 mm/Hg, or self-reported use of anti-hypertensive medication. Diabetes was defined according to American Diabetes Association guidelines [20] as an HbA_1c0_ ≥6.5 %, post-oral glucose tolerance test glucose ≥200 mg/dL, fasting glucose ≥126 mg/dL, non-fasting glucose ≥200 mg/dL, or self-reported use of anti-diabetic medication. Abdominal adiposity was defined as WC ≥102 cm for men and ≥88 cm for women. Prevalent coronary heart disease included ECG reports of possible myocardial infarction (major Q wave abnormalities and minor Q,QS waves with ST,T abnormalities) as well as self-report of heart attack or procedure (angioplasty, stent, bypass). Heart failure was self-reported, but not conformed.

### Electrocardiography

Resting, standard, 12-lead, 10 s ECGs were recorded following a standardized protocol. The participants were supine, breathing freely, and instructed not to talk during the recording. Study personnel positioned the electrodes using a chest electrode locator [21, 22]. ECGs were recorded using the GE MAC 1200 electrograph (GE, Milwaukee, Wisconsin) with a 10 mm/mV calibration at a speed of 25 mm/s. The Epidemiology Cardiology Research Center (EPICARE; Wake Forest School of Medicine, Winston Salem, NC) centrally processed the ECGs using the GE 12-SL Marquette Version 2001 (GE, Milwaukee, Wisconsin).

The automatically calculated ultra-short term, time domain measures of HRV included the median duration of the RR interval across all twelve leads as computed from heart rate, RR (ms) = 60,000 ms/heart rate (bpm); the standard deviation of all normal to normal RR intervals that indicates total HRV, SDNN $({\text{ms}}) = \left\{ {{{\left[ {\sum {_{{{\text{j}} = 1}}^{\text{n}} \left( {{\text{RR}}_{\text{mean}} - {\text{RR}}_{\text{j}} } \right)^{2} } } \right]} \mathord{\left/ {\vphantom {{\left[ {\sum {_{{{\text{j}} = 1}}^{\text{n}} \left( {{\text{RR}}_{\text{mean}} - {\text{RR}}_{\text{j}} } \right)^{2} } } \right]} {\left( {{\text{n}} - 1} \right)}}} \right. \kern-0pt} {\left( {{\text{n}} - 1} \right)}}} \right\}^{0.5}$ and the root mean square of successive differences in all normal to normal RR intervals that indicates the degree the RR interval changes between adjacent beats, ${\text{RMSSD}}\left( {\text{ms}} \right) = \left\{ {{{\left[ {\sum {_{{{\text{j}} = 1}}^{{^{\text{n}} }} \left( {{\text{RR}}_{\text{j + 1}} - {\text{RR}}_{\text{j}} } \right)^{2} } } \right]} \mathord{\left/ {\vphantom {{\left[ {\sum {_{{{\text{j}} = 1}}^{{^{\text{n}} }} \left( {{\text{RR}}_{\text{j + 1}} - {\text{RR}}_{\text{j}} } \right)^{2} } } \right]} {\text{n}}}} \right. \kern-0pt} {\text{n}}}} \right\}^{0.5}$.

### Statistical methods

All analyses used complex survey methods and were weighted to account for design effects, cluster sampling and the use of stratification in sample selection [18]. Means and prevalence rates are therefore presented as weighted statistics to adjust for potential bias due to differential nonresponse at the household and person levels. We estimated the mean and 95 % confidence interval (CI) of RMSSD, SDNN and RR interval by demographic characteristics, and quartiles of glucose homeostasis measures stratified by sex. Following the recommendations of Rothman [23], effect sizes and confidence intervals were presented in place of p values and significance testing.

Multivariable linear regression analyses were used to estimate associations between exposures (fasting insulin, fasting glucose, HbA_1c_, and insulin resistance computed according to the homeostasis model assessment of insulin resistance (HOMA-IR)) and outcomes (SDNN, RMSSD and RR). Separate models were constructed for each exposure-outcome pair and exposures were not adjusted for other glucose homeostasis measures. The adjustment variables considered in all models included sex, age groups (18–24, 25–34, 35–44, 45–54, 55–64, and 65+ years old), Hispanic/Latino background, field center, continuous BMI, continuous waist circumference, current smoking, heart rate modifying drugs (beta-blockers, non-hydropyridine calcium channel blockers, and sympathomimetics), and the interactions between glucose homeostasis measures with gender, age and abdominal adiposity. In sensitivity analyses, we adjusted for self-reported physical activity and alcohol consumption. For simplicity, interactions were selected based on model fit using adjusted R^2^ values for RMSSD. Interactions were selected if adjusted R^2^ values increased model fit by at least 0.001. When interaction terms were selected, we reported stratified results. For HbA_1c_, the waist circumference interaction was selected but categorized as WC ≥102 cm for men and ≥88 cm for women to indicate abdominal adiposity for ease of interpretation. For anthropometric measures, WC was chosen as a covariate based on a slightly higher adjusted R^2^ value compared with the model with BMI (difference in adjusted R^2^ ≈ 0.00013). The best fitting models for SDNN were similar and the same model was used for RR interval for comparability. Analyses were conducted using complex survey procedures in SAS (version 9.3, SAS Institute, Inc., Cary, NC) and SUDAAN (release 11.0.0, Research Triangle Institute, Research Triangle Park, NC).

## Results

About half of the HCHS/SOL target population for this study was comprised of women and the mean age was 38.8 years (Table 1). Almost 37 % of the HCHS/SOL target population was obese and 51 % had abdominal adiposity. Individuals of Mexican background were the largest Hispanic/Latino background group at 38 % of the HCHS/SOL target population. Men had an 8 % lower mean RMSSD, similar SDNN, and 4 % higher RR compared with women (Table 2). Individuals taking beta-blockers and non-hydropyridine calcium channel blockers had 35–66 % higher mean RMSSD and SDNN than non-users, but those taking beta-blockers had a 5 % lower mean RR. Mean RMSSD and SDNN was lower by 53–56 % between age groups 18–24 and 65+ years. All measures were 4–11 % lower among those without abdominal adiposity versus those with abdominal adiposity. Those with obesity had 4 % lower RR but similar RMSSD and SDNN versus non-obese individuals.

**Table 1:** Demographic characteristics

Characteristic	N	% or Mean	95 % CI
Male (%)	4833	48.11	(46.79, 49.42)
Age group (%)			
18–24	1444	18.94	(17.73, 20.21)
25–34	1819	24.63	(23.25, 26.06)
35–44	2461	23.03	(21.81, 24.30)
45–54	3562	18.01	(17.03, 19.02)
55–64	2106	10.34	(9.61, 11.12)
65+	602	5.05	(4.47, 5.70)
Hispanic background (%)			
Mexican/Mexican American	4784	38.13	(34.90, 41.46)
Puerto Rican	1809	15.09	(13.58, 16.74)
Cuban	1753	19.61	(16.54, 23.08)
Dominican	1084	10.02	(8.68, 11.55)
Central American	1320	7.59	(6.48, 8.86)
South American	854	5.28	(4.63, 6.02)
Other	390	4.28	(3.68, 4.96)
Body mass index (BMI; kg/m^2^)		28.88	(28.68, 29.08)
Waist circumference (cm)		95.90	(95.44, 96.36)
Obese (BMI ≥ 30 kg/m^2^)	4597	36.86	(35.37, 38.38)
Abdominal adiposity	6818	51.44	(49.82, 53.05)
Systolic blood pressure (mmHg)		118.32	(117.85, 118.79)
Diastolic blood pressure (mmHg)		71.79	(71.43, 72.15)
Fasting glucose (mg/dL)		93.51	(93.28, 93.74)
Fasting insulin (mU/L)		12.06	(11.78, 12.34)
HbA_1c_ (%)		5.41	(5.40, 5.42)
HOMA-IR (index)		2.83	(2.76, 2.90)
Current smoking	2382	21.86	(20.60, 23.17)
Coronary heart disease	416	3.19	(2.71, 3.75)
Self-reported heart failure	132	0.87	(0.68, 1.10)
Beta blocker use	520	3.24	(2.86, 3.66)
Non-hydropyridine calcium channel blocker use	300	1.98	(1.63, 2.41)
Sympathomimetic medication use	441	3.50	(3.01, 4.07)

The Hispanic Community Health Study/Study of Latinos (HCHS/SOL) baseline examination (2008–2011)

Abdominal adiposity (waist circumference ≥102 cm for men and ≥88 cm for women)

**Table 2:** Mean and 95 % confidence interval (CI) of RMSSD, SDNN and RR interval by demographic characteristics

Characteristic	RMSSD (ms)		SDNN (ms)		RR (ms)	
	Mean	95 % CI	Mean	95 % CI	Mean	95 % CI
Sex (%)						
Male	40.72	(39.07, 42.38)	34.20	(32.98, 35.43)	1010	(1003, 1016)
Female	44.43	(43.08, 45.78)	35.34	(34.36, 36.32)	968	(963, 973)
Age group (%)						
18–24	63.33	(59.82, 66.85)	49.20	(46.76, 51.64)	981	(971, 991)
25–34	50.37	(48.22, 52.52)	40.30	(38.61, 41.98)	988	(978, 998)
35–44	37.56	(36.14, 38.97)	31.41	(30.27, 32.55)	985	(976, 993)
45–54	30.32	(29.38, 31.26)	26.13	(25.39, 26.87)	994	(987, 1001)
55–64	26.51	(25.39, 27.63)	23.59	(22.59, 24.58)	992	(984, 1001)
65+	27.59	(24.63, 30.54)	23.20	(20.71, 25.68)	1006	(991, 1022)
Obese (BMI ≥ 30 kg/m^2^)						
Yes	43.49	(42.09, 44.89)	35.55	(34.50, 36.61)	1001	(996, 1006)
No	41.20	(39.33, 43.08)	33.49	(32.22, 34.76)	966	(959, 973)
Abdominal adiposity						
Yes	44.50	(42.92, 46.08)	36.64	(35.44, 37.83)	1010	(1004, 1016)
No	40.89	(39.37, 42.42)	33.05	(31.98, 34.13)	967	(962, 973)
Current smoking						
Yes	42.07	(40.96, 43.17)	34.22	(33.44, 35.01)	985	(981, 990)
No	44.72	(41.75, 47.68)	36.83	(34.63, 39.02)	998	(988, 1009)
Beta blocker use						
Yes	43.14	(41.97, 44.30)	35.24	(34.40, 36.07)	987	(982, 991)
No	31.91	(28.80, 35.01)	24.84	(22.65, 27.03)	1042	(1024, 1060)
Non-hydropyridine calcium channel blocker use						
Yes	43.11	(41.96, 44.27)	35.16	(34.33, 35.98)	989	(984, 993)
No	25.92	(22.42, 29.42)	22.14	(19.73, 24.56)	981	(959, 1003)
Sympathomimetic medication use						
Yes	42.76	(41.62, 43.91)	34.88	(34.07, 35.69)	990	(986, 995)
No	43.04	(33.48, 52.59)	35.45	(28.13, 42.78)	936	(917, 954)

*CI* confidence interval, *RMSSD* root mean square of successive differences in RR intervals*, SDNN* standard deviation of all normal to normal RR intervals*, RR* median duration of the RR interval*, BMI* body mass index

Abdominal adiposity (waist circumference ≥102 cm for men and ≥88 cm for women)

**Fig. 1:**
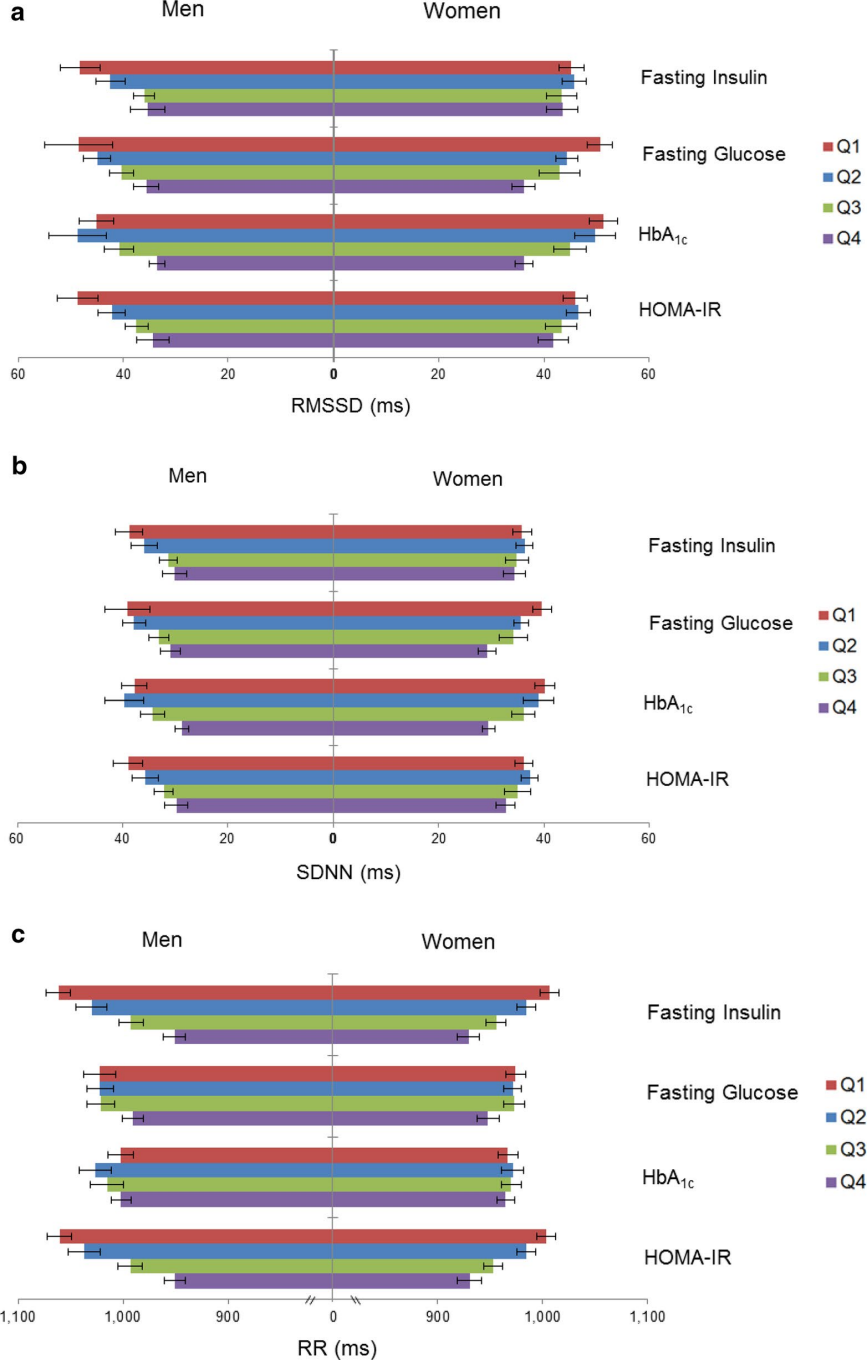
Mean (95 % confidence interval) of **a** RMSSD, **b** SDNN, and **c** RR by quartiles (*Q*) of glycemic homeostasis marker stratified by sex. Values are *Q1* (lowest) to *Q4* (highest). *RMSSD* root mean square of successive differences in RR intervals*, SDNN* standard deviation of all normal to normal RR intervals, RR median duration of the RR interval; *HbA*_*1c*_ glycated hemoglobin, *HOMA*-*IR* homeostasis model assessment of insulin resistance

HRV and RR varied by quartiles of glucose homeostasis measures (Fig. 1). Trends for RMSSD and SDNN were very similar. Comparing those in quartile 4 to those in quartile 1 for fasting insulin and HOMA-IR, the mean RMSSD was 27 and 29 % lower, respectively, among men. But for women, the mean differences were much smaller (4 and 9 %, Fig. 1). Comparing those in quartile 4 to those in quartile 1 of fasting glucose, the mean RMSSD was 27 % lower in men and 29 % lower in women. Trends for SDNN were similar to those of RMSSD. Subgroup trend differences for RR interval were much less pronounced. For example, comparing those in quartile 4 to those in quartile 1 of fasting insulin, the mean RR interval was lower by 10 % in men and 8 % in women.

After adjustment for gender, age, Hispanic/Latino background, field center, and medication use, the association of fasting glucose with RMSSD was greatly reduced (from −0.65 to −0.20 mm change in RMSSD for each mg/dL increase in fasting glucose; Table 3). The association of fasting glucose with RR was more pronounced after adjustment for all covariates (β = −1.55 vs −0.80). Fasting insulin was negatively associated with all HRV measures, and the association was stronger among men compared with women. The multivariable models for fasting insulin were similar to unadjusted models (β = −0.45 vs β = −0.44 for men, β = −0.06 vs β = −0.14 for women). HbA_1c_ was negatively associated with RMSSD among individuals with abdominal adiposity, defined by sex-specific cut-points for WC, but not among those without abdominal adiposity. While the unadjusted subgroup effects for HbA_1c_ were similar for individuals with and without abdominal adiposity, after adjusting for gender, age, Hispanic/Latino background, field center, and medication use, differences in subgroups emerged for all HRV measures. The association of HbA_1c_ and SDNN were similar to that of RMSSD. After adjustment, there was little or no association between HbA_1c_ and RR among those without abdominal adiposity, but there was a 22.9 ms decrease in RR for each unit increase in HbA_1c_ among those with abdominal adiposity. As with fasting insulin, HOMA-IR was associated with all HRV measures in both men and women, but the association was slightly stronger among men. Adjusting for physical activity and current weekly alcohol consumption did not alter the results (data not shown).

**Table 3:** Adjusted associations of glucose homeostasis markers with RMSSD, SDNN and RR interval

Outcome/exposure	Unadjusted		Model 1^a^		Model 2^b^	
	β	95 % CI	β	95 % CI	β	95 % CI
RMSSD (ms)						
Fasting glucose (mg/dL)	−0.65	(−0.77, −0.53)	−0.20	(−0.33, −0.07)	−0.16	(−0.28, −0.05)
Fasting insulin (mU/L), men	−0.45	(−0.66, −0.24)	−0.43	(−0.62, −0.24)	−0.44	(−0.62, −0.26)
Fasting insulin (mU/L), women	−0.06	(−0.20, 0.08)	−0.13	(−0.24, −0.01)	−0.14	(−0.27, −0.01)
HbA_1c_, no abdominal adiposity	−12.8	(−15.83, −9.78)	3.61	(0.26, 6.97)	3.22	(−0.09, 6.53)
HbA_1c_, abdominal adiposity	−16.5	(−20.09, −13.04)	−3.57	(−6.74, −0.41)	−3.62	(−6.75, −0.48)
HOMA-IR, men	−1.88	(−2.71, −1.05)	−1.65	(−2.37, −0.92)	−1.66	(−2.34, −0.97)
HOMA-IR, women	−0.49	(−1.03, 0.06)	−0.55	(−1.03, −0.07)	−0.58	(−1.10, −0.06)
SDNN (ms)						
Fasting glucose (mg/dL)	−0.43	(−0.52, −0.34)	−0.13	(−0.23, −0.04)	−0.10	(−0.20, −0.01)
Fasting insulin (mU/L), men	−0.29	(−0.44, −0.15)	−0.28	(−0.41, −0.15)	−0.26	(−0.38, −0.13)
Fasting insulin (mU/L), women	−0.05	(−0.15, 0.05)	−0.10	(−0.19, −0.01)	−0.08	(−0.18, 0.02)
HbA_1c_, no abdominal adiposity	−9.11	(−11.44, −6.78)	2.29	(−0.20, 4.77)	1.98	(−0.48, 4.44)
HbA_1c_, abdominal adiposity	−11.8	(−14.42, −9.26)	−2.56	(−4.94, −0.19)	−2.60	(−4.96, −0.23)
HOMA-IR, men	−1.23	(−1.79, −0.67)	−1.07	(−1.56, −0.58)	−0.97	(−1.44, −0.49)
HOMA-IR, women	−0.37	(−0.77, 0.03)	−0.43	(−0.79, −0.06)	−0.35	(−0.77, 0.06)
RR (ms)						
Fasting glucose (mg/dL)	−0.80	(−1.29, −0.31)	−2.02	(−2.52, −1.52)	−1.55	(−2.06, −1.04)
Fasting insulin (mU/L), men	−4.41	(−5.13, −3.69)	−4.39	(−5.11, −3.67)	−4.09	(−4.90, −3.29)
Fasting insulin (mU/L), women	−3.15	(−3.94, −2.36)	−3.07	(−3.80, −2.34)	−2.82	(−3.54, −2.09)
HbA_1c_, no abdominal adiposity	19.58	(2.84, 36.32)	1.03	(−16.54, 18.61)	−0.33	(−17.83, 17.18)
HbA_1c_, abdominal adiposity	−8.18	(−20.46, 4.11)	−22.8	(−35.12, −10.55)	−22.9	(−35.24, −10.72)
HOMA-IR, men	−17.2	(−20.07, −14.46)	−17.3	(−20.15, −14.47)	−16.0	(−19.18, −12.93)
HOMA-IR, women	−12.8	(−15.70, −10.03)	−12.6	(−15.32, −10.03)	−11.5	(−14.17, −8.91)

Abdominal adiposity (waist circumference ≥102 cm for men and ≥88 cm for women)

*RMSSD* root mean square of successive differences in RR intervals*, SDNN* standard deviation of all normal to normal RR intervals*, RR* median duration of the RR interval, *HbA*_*1c*_ glycated hemoglobin, *HOMA-IR* homeostasis model assessment of insulin resistance

^a^Model 1 includes gender, age groups (each as an indicator variable), Hispanic background, and medications: β-blocker, non-hydropyridine calcium channel blocker, sympathomimetics, and field center

^b^For fasting glucose, fasting insulin, and HOMA-IR, model 2 includes model 1 plus current smoking and waist circumference. For HbA_1C_, model 2 includes model 1 plus current smoking

## Discussion

Our study contributes new information to the field by evaluating the cross-sectional association of multiple glucose homeostasis measures with cardiac autonomic function in a diverse population of Hispanic/Latino adults. In this cross-sectional study, higher fasting glucose was associated with lower HRV, as assessed by several HRV measures, in all subgroups; higher fasting insulin and HOMA-IR were associated with lower HRV particularly in men; and higher HbA_1c_ was associated with lower HRV in individuals with abdominal adiposity. These results suggest that reduced cardiac autonomic function occurs among those with metabolic impairments even before the onset of overt diabetes. The association was more pronounced among men and those with abdominal adiposity, defined by sex-specific cut-points for waist circumference.

### Prior studies of glucose homeostasis measures and cardiac autonomic function

Our findings are in agreement with prior studies among people without diabetes showing associations between glucose homeostasis measures with reduced cardiac autonomic function, although results are inconsistent across glucose homeostasis measures [4, 5, 9, 13]. The atherosclerosis risk in communities study reported weak, inverse cross-sectional associations between fasting glucose and insulin quintiles with RMSSD and RR among people without diabetes [9]. Insulin quintiles were negatively associated with SDNN, whereas no associations were seen between glucose quintiles and SDNN. In contrast, fasting glucose was negatively associated with SDNN and positively associated with heart rate, but not associated with RMSSD in a study among participants with normal fasting glucose levels [5]. In a healthy cohort free of diabetes, fasting insulin and fasting glucose levels were associated with reduced cardiac autonomic function measured by baroreflex sensitivity, but the association with fasting glucose was no longer significantly associated after adjusting for age, blood pressure and body mass index [13]. The patterns as reported above include HRV measured from 2 min to 24 h ECGs, and are similar to our results from 10 s ECGs. However, both fasting insulin and fasting glucose levels were associated with lower RMSSD, SDNN and RR in this study, with stronger associations among subgroups.

We add to the literature by reporting on the associations between HbA_1c_ and HOMA-IR with reduced heart rate variability. A study among participants with normal fasting glucose levels showed that only HbA_1c_ was associated with reduced cardiac autonomic function defined as the lowest 25th percentile for SDNN and baroreflex sensitivity [4]. No associations were observed between fasting insulin and fasting glucose with SDNN and mean NN (RR interval). We found that HbA_1c_ was associated with RMSSD, SDNN and RR among those with abdominal adiposity (WC ≥102 cm for men and ≥88 cm for women), but no associations were observed among individuals with waist circumferences below the sex-specific cut-points. Discrepancies in the association between measures of glucose metabolism and HRV between prior studies and our results could be related to differences in HRV methodology and the study populations since previous studies involved mostly older Caucasian participants. Additionally, there were no reports of whether the investigators examined effect modification by sex or adiposity.

### Potential mechanisms of the sex differences in glucose homeostasis and cardiac autonomic function

There was a stronger association between fasting insulin and HOMA-IR with HRV among men compared with women. Sex differences in HRV among those without diabetes have not been well studied, but evidence among those with diabetes suggests that there are sex differences in nerve conduction [24]. Men tend to present symptoms of diabetic neuropathy earlier than women [25], and might be more susceptible to autonomic failure (as defined by e.g. low HRV) compared with women [26, 27]. These observations indicate that men have earlier or stronger manifestations of reduced cardiac autonomic function compared with women, which could explain the effect modification by sex observed in this study.

### Abdominal adiposity in cardiac autonomic function

HbA_1c_ was only associated with HRV measures among those with abdominal adiposity defined as a high waist circumference. This is consistent with previous studies showing that visceral, or central, adipose tissue is more strongly associated with metabolic dysregulation, muscle sympathetic nerve activity [28, 29], autonomic nervous system activity [30], and HR [31]. The adipokine leptin is associated with autonomic dysfunction [32, 33], and may be stronger among those with higher levels of adiposity [34]. HbA_1c_ is a time-integrated marker of long-term glucose exposure, thus the observed association suggests that those with abdominal obesity and poor glycemic control are prone to have lower cardiac autonomic function, although we were not able to evaluate such a complex relationship in this cross-sectional analysis.

### Glucose homeostasis measures and modulation of cardiac autonomic function

The observed associations between glucose homeostasis measures with SDNN, RMSSD and RR suggest that insulin resistance and impaired glycemic control adversely affect autonomic function; however, the temporal relationship between insulin resistance and poor glucose control with reduced cardiac autonomic activity has not been elucidated. Several mechanisms have been implicated in the link between glucose homeostasis measures with reduced autonomic function. Insulin resistance could cause reduced autonomic function by triggering sympathetic nervous system activity [35, 36], suppressing vagal cardiac activity [37], and regulating energy balance and homeostasis [38–41]. An enhanced formation of advanced glycation end products and their associated cross-linking molecules [42] is also reported to be associated with autonomic nerve impairment and neuropathy in individuals with diabetes [43]. Although the mechanisms that contribute to reduced cardiac autonomic function are not fully characterized, the evidence above indicates that insulin resistance and glucose metabolism are central factors.

## Limitations

Our study had limitations that should be noted. We used ultra-short HRV measures without control for respiration. Although guidelines from the Task Force of the European Society of Cardiology the North American Society of Pacing Electrophysiology recommend a 5-min recording for time domain HRV indices [19], ultra-short term measures of HRV have been shown to have high reproducibility [44] and their predictive validity has been described [45–50]. Time limitations precluded the acquisition of ECG records of longer duration, therefore only time domain indices were available. Thus, frequency domain indices to distinguish parasympathetic and sympathetic modulation of cardiac autonomic activity and geometric measures that would allow for the assessment of different geometrical forms were not attempted. Lastly, we are unable to address the temporality of the observed associations given the cross-sectional nature of these analyses.

## Conclusions

A comprehensive set of glucose homeostasis measures were associated with reduced autonomic activity in some subgroups of Hispanic/Latino adults without diabetes. The associations were stronger among men and in those with abdominal adiposity, offering clues for the pathophysiologic processes involved as well as opportunity for identification of those at high risk before autonomic control is manifestly impaired. While screening for reduced cardiac autonomic function is recommended for risk assessment in persons with diabetes, our results suggest that impairment in glucose homeostasis is associated with lower autonomic activity even before diabetes becomes clinically manifest. The longitudinal follow-up of this population can be expected to offer insights into the temporal relationships of glucose homeostasis measures with HRV, and its utility in risk stratification.

## Abbreviations

BMI: body mass index
CI: confidence interval
CVD: cardiovascular disease
ECG: electrocardiogram
HbA_1c_: glycated hemoglobin
HCHS/SOL: The Hispanic Community Health Study/Study of Latinos
HOMA-IR: homeostasis model assessment of insulin resistance
HRV: heart rate variability
RMSSD: root mean square of successive differences in RR intervals
RR: RR interval
SDNN: standard deviation of all normal to normal RR intervals
WC: waist circumference
